# Competitive Deep-Belief Networks for Underwater Acoustic Target Recognition

**DOI:** 10.3390/s18040952

**Published:** 2018-03-23

**Authors:** Honghui Yang, Sheng Shen, Xiaohui Yao, Meiping Sheng, Chen Wang

**Affiliations:** School of Marine Science and Technology, Northwestern Polytechnical University, 127 West Youyi Road, Beilin District, Xi’an 710072, China; shensheng@mail.nwpu.edu.cn (S.S.); yaoxiaohui@mail.nwpu.edu.cn (X.Y.); smp@nwpu.edu.cn (M.S.); wingbuaa@163.com (C.W.)

**Keywords:** underwater acoustics, machine learning, deep learning, hydrophone

## Abstract

Underwater acoustic target recognition based on ship-radiated noise belongs to the small-sample-size recognition problems. A competitive deep-belief network is proposed to learn features with more discriminative information from labeled and unlabeled samples. The proposed model consists of four stages: (1) A standard restricted Boltzmann machine is pretrained using a large number of unlabeled data to initialize its parameters; (2) the hidden units are grouped according to categories, which provides an initial clustering model for competitive learning; (3) competitive training and back-propagation algorithms are used to update the parameters to accomplish the task of clustering; (4) by applying layer-wise training and supervised fine-tuning, a deep neural network is built to obtain features. Experimental results show that the proposed method can achieve classification accuracy of 90.89%, which is 8.95% higher than the accuracy obtained by the compared methods. In addition, the highest accuracy of our method is obtained with fewer features than other methods.

yellow

## 1. Introduction

A passive sonar system is the main equipment for identifying underwater or surface targets through their radiated noise. Ship-radiated noise signals are produced by ships during operation, and they are acquired by hydrophones. Because of the convolution effect between underwater channels, the processing of received underwater acoustic signals is extremely complex. Hence, underwater acoustic target recognition still depends on the decision of well-trained sonarmen, which can be highly inaccurate due to the need of continuous monitoring, and at times much affected by weather conditions. In addition, such manual monitoring usually is affected by mood, environment and the physical condition of human beings.

To solve these problems, considerable work has been devoted to the development of underwater acoustic target automatic recognition systems. Most of the efforts focus on extracting relevant features and developing nonlinear classifiers using these features. The extracted underwater acoustic target features mainly include waveform features, wavelet features and auditory features. In the field of time-domain waveform analysis, zero-crossing features and peak-to-peak amplitude features were presented to describe rotation of a propeller by Meng [1,2]. However, their performance was greatly reduced under noisy environments. Azimi-Sadjadi [3] studied features extracted based on wavelet packets. Wei [4] classified underwater acoustic targets by combining wavelet features and principle component analysis (PCA). In there, it was found that generally there is difficulty in determining decomposition series of wavelets due to lack of prior knowledge. In recent years, an auditory model was proposed to extract features using the human hearing system as a reference. Auditory features based on dissimilarity evaluation were put forward by Yang [5]. In [6], Zhang utilized Mel-frequency cepstral coefficients (MFCCs) to describe underwater acoustic targets. However, their frequency resolution was too low to describe details of the spectrum. Tuma [7] discussed the fusion of multi-domain features and joint optimization of classifiers. Yang [8] presented an integrated recognition system using feature fusion, feature selection and a support vector machine (SVM). A common shortcoming of all these traditional approaches discussed above is that they used shallow classifiers on top of hand-engineered features. This requires a considerable amount of engineering skill, domain expertise and prior knowledge to extract good features. However, it is very difficult to obtain enough prior knowledge of underwater acoustic targets in complex ocean environments. The performance of these traditional methods is not satisfactory. Due to the complexity of this task, automatic recognition of passive sonar signals is still a difficult task at present.

In recent years, deep-learning approaches have been studied as a way of building hierarchical representations from original data. The key advantage of deep learning is that it can learn features automatically using a general-purpose learning procedure without prior knowledge. Supervised learning methods, instead of unsupervised pretraining methods, have gradually become the focus of research in the fields of image recognition and speech recognition with the increase of labeled datasets. However, large amounts of labeled data are required by supervised deep-learning models, while it is very difficult to obtain labeled data in the field of underwater acoustic target recognition. Underwater target recognition can be considered as a problem of small sample size. By contrast, as mentioned in [9], unsupervised pretraining models such as deep-belief networks (DBNs) can make use of unlabeled data, and only a small amount of labeled data is required. Therefore, DBNs are more suitable for underwater acoustic target recognition. Unlabeled underwater acoustic data, such as ocean ambient noise and ship-radiated noise, can be obtained much more easily compared with labeled data. DBNs can learn basic concepts of underwater acoustic signals with a large amount of unlabeled data, and then improve the classification performance of learned deep features with a small amount of labeled data.

Several underwater acoustic target-recognition methods based on DBN have been proposed in our previous papers. In [10], unsupervised pretraining and group sparse punitive functions were integrated as a hyper-regularization method to initialize the network parameters. In [11], an unsupervised grouping method based on mutual information between features learned from ship-radiated noise was proposed to improve the classification ability of features. However, both grouping methods in [10,11] did not directly consider the relationship between deep features and categories.

In this paper, we propose a new DBN method called competitive deep-belief networks (CDBNs) for underwater acoustic target recognition. The main idea of the proposed method is: (1) pretraining the DBN with a large amount of unlabeled data in an unsupervised manner to learn basic concepts of underwater acoustic signals; (2) grouping deep features according to their relevance with categories; (3) using competitive learning to enhance discriminating information of deep features among groups; (4) fine-tuning the CDBN with a small number of labeled data.

The contributions of this paper are briefly summarized as follows:

1. The proposed CDBN method integrates a new competitive learning mechanism into deep-belief networks to learn more robust and discriminative features for underwater acoustic target recognition.
2. The proposed CDBN method can make use of unlabeled samples to solve the small-sample-size problem of underwater acoustic target recognition.
3. Compared with traditional hand-engineered feature-extraction methods, the proposed method can learn features from datasets automatically, and does not require prior knowledge.
4. The experimental results demonstrated that the proposed CDBN method is effective for underwater acoustic target recognition. It can significantly reduce the random noise and enhance the line-spectrum characteristics of ship noises, and the CDBN features have better classification performance than other hand-engineered features.

This paper is organized as follows. A brief description of the proposed CDBN is given in Section 2 first. Relevant issues about the design of the CDBN system are discussed in Section 3. Section 4 shows the experiment results and relevant discussions, followed by the conclusions in Section 5.

## 2. Competitive Deep-Belief Networks

**Figure 1:**
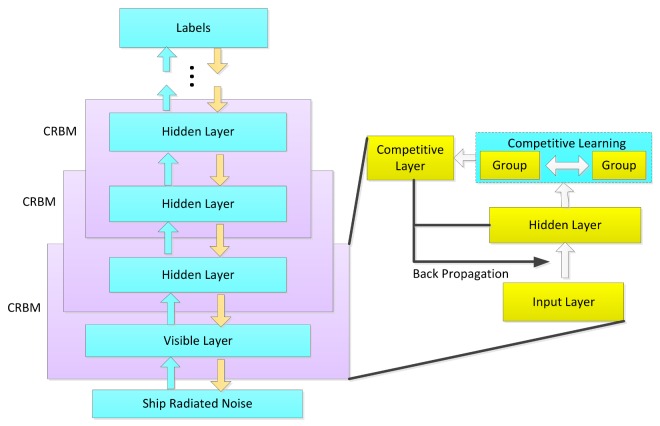
The structure of CDBN.

The framework of the CDBN is shown in Figure 1. The training procedure is described as follows. Firstly, an RBM is pretrained with a large amount of unlabeled data and a small number of labeled data in an unsupervised learning way, which provides initialized parameters for further optimization. Secondly, for the hidden layer, this architecture groups its hidden units based on their scores corresponding to different categories. Thirdly, a competitive layer with a mechanism of intragroup enhancement and intergroup inhibition is constructed by adding a lateral connection among the grouped hidden units. A gradient algorithm is applied to update parameters of RBM to build the competitive restricted Boltzmann machine (CRBM). Finally, the CDBN is constructed by fitting a stack of the designed CRBM, and the whole model is then discriminatively fine-tuned to maximize its probability of predicting the underwater acoustic targets.

## 3. Competitive Deep-Belief Network Design

### 3.1. Restricted Boltzmann Machine

RBM is a stochastic neural network with a visible layer and a hidden layer. The visible units $v={\left(v_{1},v_{2},\ldots ,v_{n}\right)}^{T}$ that represent observations are connected to hidden units $h={\left(h_{1},h_{2},\ldots ,h_{m}\right)}^{T}$ that represent features by using undirected weighted connections. To deal with real-valued ship-radiated noise, Gaussian–Bernoulli RBM (GB–RBM) is used, in which the hidden units are binary while the input units are linear with Gaussian noise. The weights and biases define a probability distribution over the joint states of the visible and hidden units via the energy function [12,13,14]:

(1) $$E\left(v,h|\theta \right)=\sum_{i=1}^{n}\frac{{\left(v_{i}-a_{i}\right)}^{2}}{2}-\sum_{j=1}^{m}b_{j}h_{j}-\sum_{i=1}^{n}\sum_{j=1}^{m}v_{i}W_{ij}h_{j},$$

where $\theta =(W_{ij},a_{i},b_{j})$ , weight $W_{ij}$ represents the symmetric interaction term between visible unit *i* and hidden unit *j*, and $a_{i}$ and $b_{j}$ are biases terms. *n* and *m* are numbers of visible and hidden units, respectively.

The conditional distribution P(h|v,θ) is:

(2) $$P\left(h_{j}=1|v,\theta \right)=\sigma \left(b_{j}+\sum_{i}v_{i}W_{ij}\right),$$

where $\sigma \left(x\right)=1/\left(1+e^{-x}\right)$.

Likewise, the conditional distribution P(v|h,θ) is:

(3) $$P\left(v_{i}=1|h,\theta \right)=N\left(a_{j}+\sum_{j}h_{j}W_{ij},1\right),$$

where N(μ,V) obeys the Gaussian distribution with mean μ and variance *V*.

The gradient of the log probability of the training data is:

(4) $$\frac{\partial lnp(v)}{\partial \theta }=E_{h|v}\left[\frac{\partial E(v,h)}{\partial \theta }\right]-E_{h,v}\left[\frac{\partial E(v,h)}{\partial \theta }\right],$$

where E is the expectation.

By approximating the expectations by the contrastive divergence (CD) algorithm, the gradient can be calculated effectively. Given the training data is conditioned to the visible units, the conditional independence of hidden units makes it easy for RBM to extract features in the posterior distribution. Training neutral networks with limited labeled data is a nonconvex optimization problem. However, it is often easy to obtain a large amount of unlabeled ship-radiated noise that shares several features with labeled data from the classification task of interest. The nonconvex optimization problem can be solved by conducting unsupervised pretraining [15]. Unsupervised pretraining is implemented by a single-layer RBM that takes the entire unlabeled and labeled data as input. The unsupervised training phase of RBM is designed to increase the likelihood of training data [16]. To reconstruct ship-radiated noise accurately, the hidden units of RBM must contain information about aspects of the data that are not relevant to its classification. To reduce this unwanted effect, competitive learning is introduced to improve the recognition performance.

### 3.2. Competitive Groups

Basically, RBM is a stochastic system for modeling the underlying probability distribution of a given dataset. When RBM has learned the distribution of ship-radiated noise properly, a part of vectors with information is constrained in hidden neurons [17]. This assumes that the goal of training RBM is to express the entire input acoustic signal. The actual efficiency of training RBM depends on how well the acoustic structure of ship-radiated noise is captured. Once RBM has been well trained, the distribution of connection weight vectors can reflect the statistic property of ship-radiated noise. Inherent distribution of input vectors can be explained by capturing the statistical relevance among the weight vectors. As a result, hidden units of RBM can be grouped by category.

One simple way of grouping hidden units is to find, for a given hidden unit, an input category that gives rise to the highest activation of the unit, and then this hidden unit can be viewed as a feature-extraction unit of that category. The reasoning behind this idea is that a category to which the unit is responding maximally could be a good first-order representation of what a unit is doing [18]. Only some samples contribute to high activation of a given hidden unit; the common information of these samples in the same category needs to be determined, but it is not easy to find that by inspection. A statistical method is adopted by calculating the score of a given hidden unit driven by different categories.

The RBM with *m* hidden units can be trained by the training data with *L* categories, where $h={\left(h_{1},h_{2},\ldots ,h_{m}\right)}^{T}$ represent the hidden units and k∈(1,2,…,L) represents the category number. The activation of a given hidden unit *j* is $h_{j}\left(v,\theta \right)$, which is a function of both parameter θ and input sample v. The score of hidden unit *j* driven by the *k*th category is:

(5) $$score\left(h_{jk}\right)=\frac{1}{X_{k}}\sum_{p=1}^{X_{k}}h_{j}\left(v_{k}^{p},\theta \right)-\frac{1}{X_{/k}}\sum_{q=1}^{X_{/k}}h_{j}\left({v_{/k}}^{q},\theta \right),$$

where $v_{k}$ is a sample of the *k*th category, $X_{k}$ is the number of samples in the *k*th category, $v_{/k}$ is a sample of other categories and $X_{/k}$ is the number of its samples. This score can be described as the difference of average activation values between target samples and non-target samples. A score matrix with dimension L×m is obtained.

**Figure 2:**
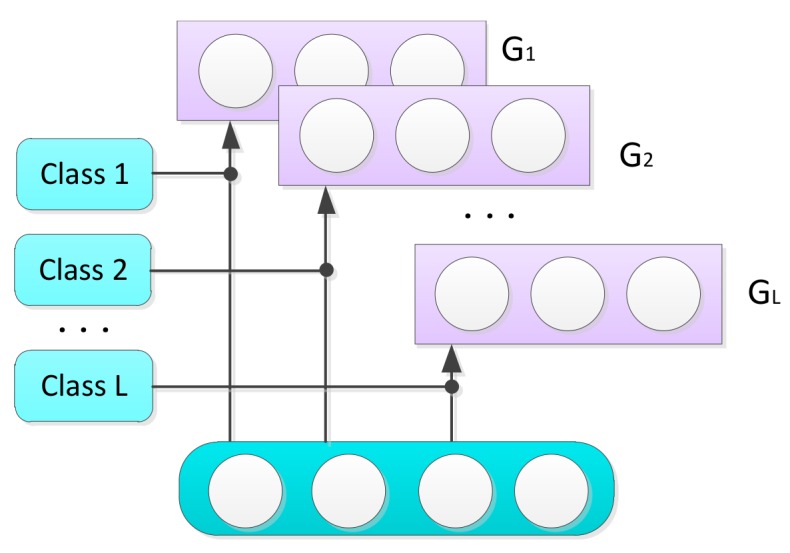
Grouping method.

The category that makes the score of a given hidden unit have a maximum value will be regarded as the corresponding category of that hidden unit. The hidden units that have the maximum score will be included in the *k*th group. The frame of the hidden-unit grouping method is shown in Figure 2. In general, each hidden unit belongs to only one group, and each group contains at least one hidden unit. Otherwise, the RBM is probably in a state of under-fitting. By applying this grouping method, the model makes the preliminary selection of hidden units and plays a role of initialization for the subsequent competitive learning.

### 3.3. Competitive Layer

**Figure 3:**
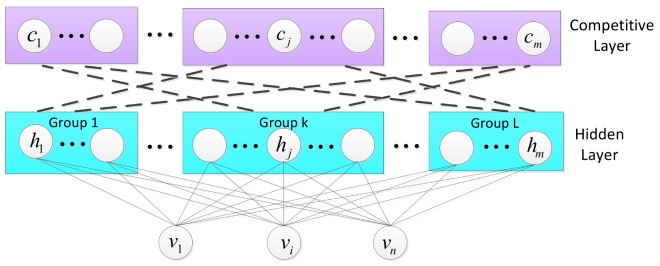
Competitive layer.

After the hidden units of RBM have been grouped as $G_{k}$, where k=1,2,…,L, the lateral connection is added between any two hidden units to build the competitive layer. The numbers of units at the input layer and competitive layer are both constant values. The competitive layer has a number of groups equal to the desired number of clusters. Each group in the competitive layer is assigned to one of the predetermined categories, and they compete with each other during training. Lateral inhibitions between groups are from small negative weights connected in units across groups. The activation function is sigmoid. Figure 3 gives a diagram of competitive layer, where the dashed lines represent negative weights. Thus it is possible to make up the competitive mechanism of intragroup enhancement and intergroup inhibition. According to the competitive mechanism, as long as the outputs of hidden units differ slightly, the network will eventually make the layer have only one activation group.

After competing with each other, the group that resembles the input vector the most wins the competition. Thus, input vector v is classified into a category to which the winner neuron belongs. To optimize the weights of RBM, a gradient-based algorithm is applied with the objective function:

(6) $$O=\frac{1}{2}\sum_{j=1}^{m}{\left[c_{j}-h_{j}\left(v\right)\right]}^{2},$$

where $c={\left(c_{1},c_{2},\ldots ,c_{m}\right)}^{T}$ is a competitive layer and $h={\left(h_{1},h_{2},\ldots ,h_{m}\right)}^{T}$ is the hidden layer.

For a single training sample v, the objective function *O* is the sum of variances of the competitive layer with the hidden layer for adapting each weight in shape to improve the extent of clustering. For each output unit *j* in the competitive layer, the desired partial derivative is:

(7) $$\frac{\partial O}{\partial W_{ij}}=\eta \left(c_{j}-h_{j}\right)h_{j}\left(1-h_{j}\right)h_{j},$$

where $c_{j}$ and $h_{j}$ represent the *j*th neurons of the competition layer and the hidden layer, respectively, and η is the learning rate. The training stops once the neurons stabilize, with each neuron in the competitive layer at the center of a cluster, and each group of such neurons mapped to a certain category. The proposed CRBM can also fulfil the task of clustering, while keeping the RBM structure unchanged.

### 3.4. Deep Architecture

After completing the training of the CRBM, the hidden units can be used as input data for training the next one. By applying such a procedure repeatedly, a CDBN with many layers is obtained to represent more complex statistical structures in ship-radiated noise. Recognition accuracy of the deep model can be further improved by discriminatively fine-tuning with a cost function that measures the discrepancy between the outputs and labels [15].

## 4. Experiments and Discussion

### 4.1. Experimental Dataset and Procedure

Experiments were conducted with sea trial data to evaluate and verify the overall performance of the proposed CDBN method. The experimental data was recorded in the South China Sea at a sampling rate of 22,050 Hz using an omnidirectional hydrophone placed at 30 m below sea level. The experimental data contains both unlabeled and labeled data. Unlabeled data includes ocean ambient noise and radiated noise from vehicles without label information. Labeled data includes radiated noise from two classes of vehicles with label information, small boat and autonomous underwater vehicle. These two classes of targets were approximately 3.5 km away from the recording hydrophone, and moved along the same route with different speeds. To avoid interference, no other ships were present within the radius of about 10 km.

There are three main sources of ship-radiated noise: internal machinery, propellers and hydrodynamic flow noise. The spectrum of ship-radiated noise comprises both discrete (linear frequency) and continuous spectrum. The discrete components of the noise spectrum are unique for each underwater acoustic target, and thus can be used to identify the target. Because ship noise has special line-spectrum characteristics, and the spectrum contains the intrinsic property of targets, the spectrum calculated by discrete Fourier transforms (DFTs) was used as input of the CDBN. Signals were divided into short frames of 186 ms, with each frame generally being considered wide-sense stationary. The spectrum of each frame or sample contains 2048 frequency bins. There were 20,000 unlabeled samples and 4000 labeled samples (2000 samples for each class) in total. In the labeled samples, 2800 samples were used for training and the rest were used for testing.

**Figure 4:**
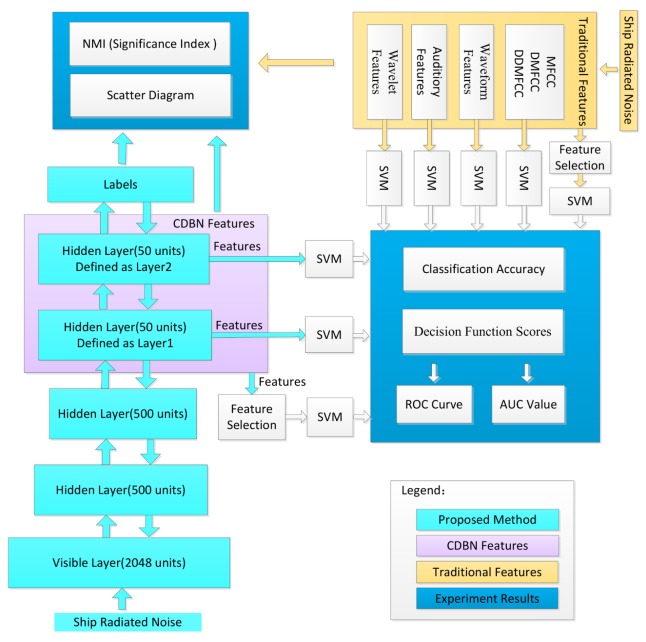
Experiment steps.

The experiment procedure is illustrated in Figure 4.

- RBM was pretrained with 20,000 unlabeled datapoints in an unsupervised manner.
- The hidden units of RBM were grouped using 2800 labeled training datapoints.
- Competitive learning was conducted to construct a CRBM.
- A 2048-500-500-50-50 CDBN was constructed by greedy layer-wise training and supervised fine-tuning to obtain CDBN features.
- SVM was used to evaluate the classification performance of CDBN features.
- The classification performance of CDBN features was compared with four widely used traditional hand-engineered feature sets.

The four feature sets were MFCC features, waveform features, auditory features and wavelet features, collectively called traditional features. MFCC features were extracted by taking the coefficients that collectively make up a Mel-frequency cepstrum. First-order differential Mel-frequency cepstrum coefficients (DMFCCs) and second-order differential Mel-frequency cepstrum coefficients (DDMFCCs) were calculated [6,19]. Waveform features were extracted via signal statistical characteristics of zero-crossing wavelength and peak-to-peak amplitude, together with their distribution [1,2]. Auditory features were extracted according to frequency division and masking properties of the human auditory system [5]. Wavelet features contained information of entropy of zero-crossing wavelength distribution density of all levels of wavelet signals and the low-frequency envelope of wavelet decomposition.

### 4.2. Grouping Experiment

**Figure 5:**
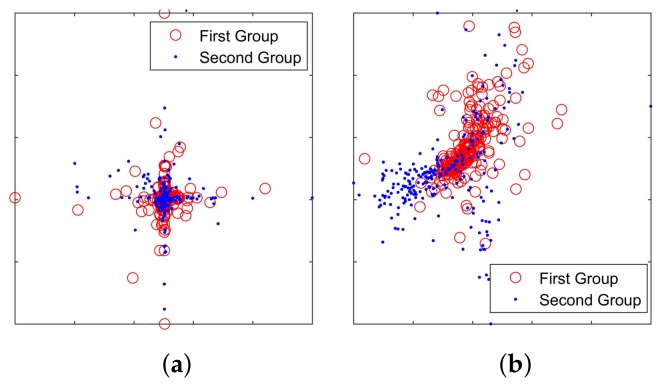
The grouped weights viewed by t-SNE. (**a**) The weights of RBM (before competition); (**b**) the weights of CRBM (after competition).

The t-SNE [20] feature-visualization method was used to observe the distribution of weight vectors in RBM and CRBM. The goal was to test whether the competitive learning can improve discriminative performance. 500 hidden units of trained RBM were divided into two groups according to categories. The scatter diagram of the grouped weight vectors of RBM viewed by t-SNE is shown in Figure 5a. A similar result of first-layer CRBM in CDBN is shown in Figure 5b, which is seen to be more distributed than that in Figure 5a. The results indicate that the proposed CRBM can learn the differences of ship types.

### 4.3. Feature Visualization

**Figure 6:**
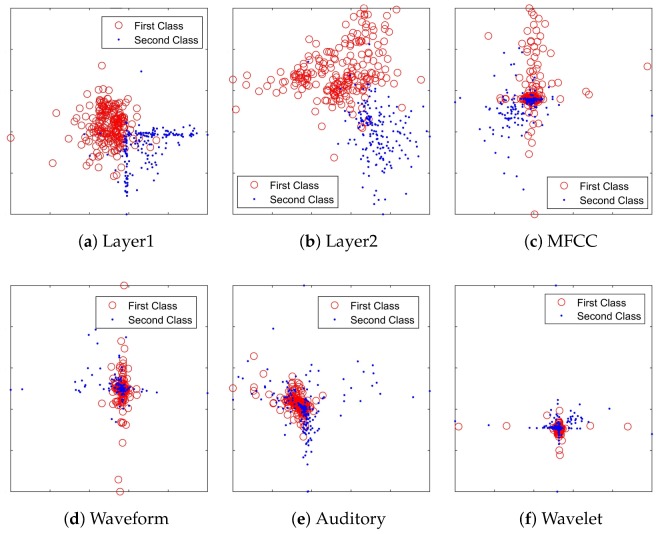
2-dimensional feature map viewed by t-SNE on the training samples. (**a**) 50 features of Layer1; (**b**) 50 features of Layer2; (**c**) 36 features of MFCC, DMFCC and DDMFCC; (**d**) 8 waveform features; (**e**) 24 auditory features; (**f**) 14 wavelet features.

The distribution of samples described by CDBN features was observed by t-SNE. 200 samples of each category were randomly selected to draw the scatter diagram. Traditional features of these samples were extracted for comparison. The last two hidden layers were extracted as the CDBN features, denoted as Layer1 and Layer2. Figure 6 shows the comparison of the scatter diagram of these samples described by CDBN features and traditional features. It is obvious that CDBN features in both Layer1 and Layer2 produce a better distribution than traditional features.

### 4.4. Features Evaluation

The significance of each feature was evaluated by calculating its relevancy with labels. Normalized mutual information (NMI) was used to measure the performance [21]. Let *F* denote the features and *L* represent the labels. NMI(F,L) is defined as:

(8) $$NMI\left(F,L\right)=\frac{H(F)-H(F|L)}{max[H(F),H(L)]},$$

where H(·) is the entropy. It is obvious that NMI(F,L) ranges from 0 to 1. NMI=1 when the two variables are identical and NMI=0 when the two variables are independent.

**Figure 7:**
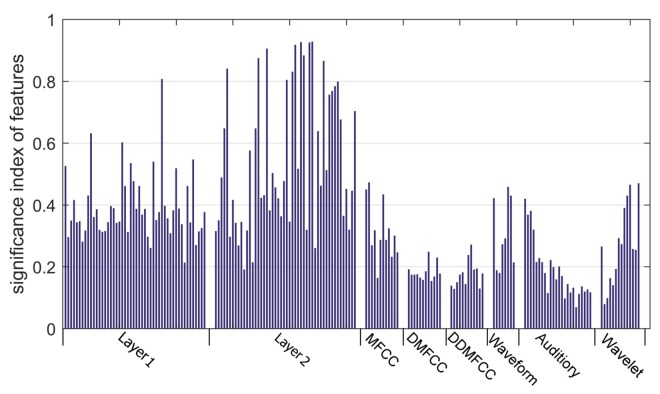
Comparison of proposed methods and traditional feature-extraction methods via significance index of features.

The significance index of all features is shown in Figure 7. The average signification index of each feature set is shown in Table 1. The average significance index of features of Layer2 is 0.554, which outperforms other features. The features of Layer1 perform second best. Features extracted by our proposed algorithm outperform all competing methods.

### 4.5. Classification Experiment

In this section, the classification performance of CDBN features and traditional features are compared. SVM classifiers were used to determine how well each category departs from others. Parameters of the classifiers were selected by using 10-fold cross-validation. Average classification accuracy over 10 random trials is reported. Results are shown in Table 1. The features of Layer1 give a classification accuracy of 80.6%, which performs better than traditional features. The classification accuracy of features of Layer2 is 86.7%, which is significantly higher than the accuracy obtained by all other features.

**Figure 8:**
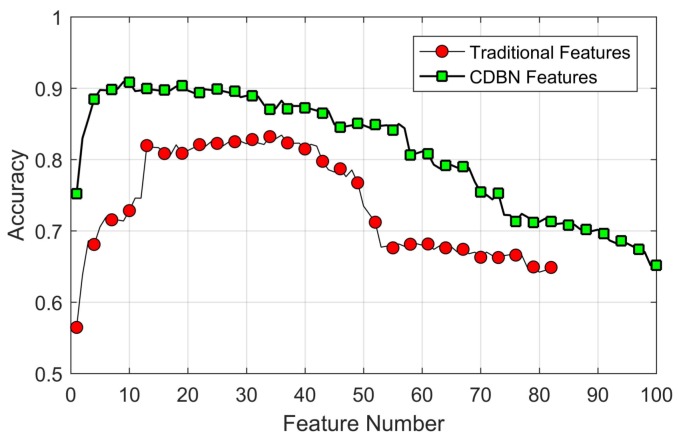
Results of feature selection on the combination of traditional features and the combination of CDBN features.

In addition, the combination of all traditional features and the combination of all hidden layers are compared. In consideration of the difference of dimension, a feature-selection algorithm was applied to the two feature sets to make a fair comparison. According to the significant index of each feature shown in Figure 8, features were sorted in descending order, and then they were selected incrementally to make up the input feature subsets of the SVM classifier.

Results are shown in Figure 8. By applying the feature-selection algorithm on traditional features, the SVM classifier could achieve an accuracy of 83.42% with 36 features, while the highest accuracy obtained on CDBN features is 90.89% with nine features. The proposed method can achieve an 8.95% improvement of the classification accuracy on fewer features.

**Figure 9:**
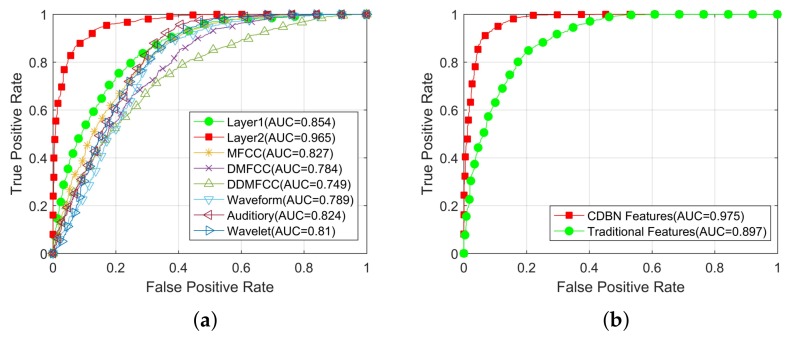
ROC curves of the proposed method and its competitors. (**a**) ROC curves obtained from features in Layer1 and Layer2 together with each traditional feature set. (**b**) ROC curves of the best 9 CDBN features and the best 36 traditional features.

Assuming that the first class was positive and the second class was negative, receivers operating characteristic (ROC) curves were constructed from decision function scores of SVM classifiers obtained on test data. Figure 9 shows the comparison of ROC curves of SVM trained on CDBN features and the previous state-of-the-art features. ROC curves obtained from features in Layer1 and Layer2 together with each traditional feature set are shown in Figure 9a. In addition, ROC curves obtained from the best nine CDBN features and the best 36 traditional features are shown in Figure 9b. Normalized area-under-ROC curve (AUC) is calculated. As shown in Figure 9a, SVM trained on features in Layer2 has the highest AUC. The performance of Layer1 is the second best. In Figure 9b, the AUC obtained from CDBN features is higher than that obtained from traditional features. In particular, the SVM trained on CDBN features exhibits 100% true-positive rate at 27.5% false-positive rate, which is lower than that trained on traditional features, and this property is desired in ship-radiated noise recognition tasks.

### 4.6. Spectrum Reconstruction of Ship-Radiated Noise with CDBN

**Figure 10:**
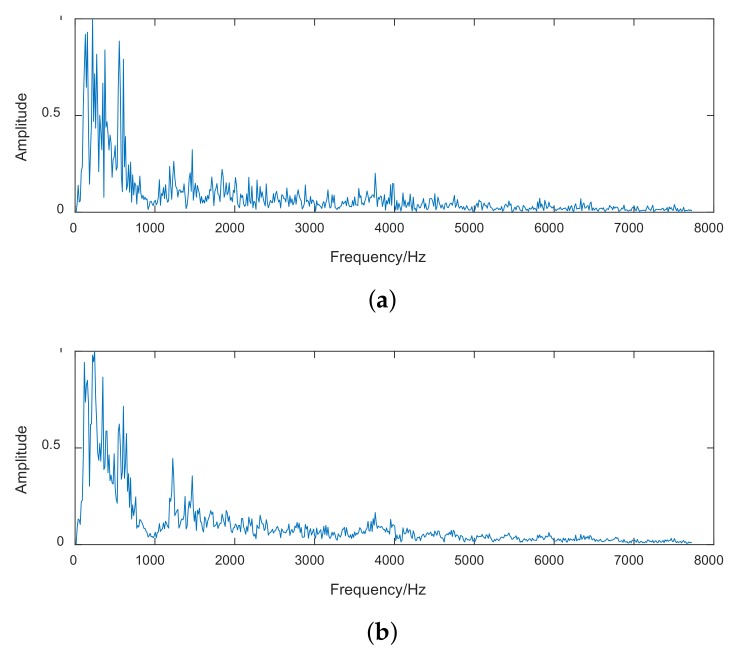
(**a**) Spectrum of ship-radiated noise reconstructed by CDBN; (**b**) Spectrum of ship-radiated noise.

As mentioned in Section 4.1, the intrinsic property of ships can be expressed by the discrete components of the spectrum of ship-radiated noise that are unique for each underwater acoustic target. Figure 10 depicts the original spectrum and reconstruction spectrum of the small-boat noise obtained by the first layer of CDBN. Frequency range below 7800Hz is shown for better viewing. It is obvious that the random noise was significantly reduced, and the line-spectrum characteristics of ships were enhanced by the CDBN method. Thus the deeper layer of CDBN can focus on the line-spectrum characteristics of ship noise, and extract features that have more classification information. As shown in Figure 6, Figure 7 and Table 1, the CDBN features outperform traditional features, and can get better classification performance.

## 5. Conclusions

A deep-learning architecture competitive deep-belief network is presented by stacking the proposed competitive restricted Boltzmann machine, which adjusts the activation level of the grouped hidden units by competitive learning. It is found that the deep-belief network pretrained with a large amount of unlabeled ship-radiated noise can solve the problem of the lack of training samples. By introducing competitive learning, the features learned by the deep-belief network have the self-clustering property. Compared with the traditional features, the competitive deep-belief network features have a greater relevance with the labels. By implementing our algorithm on the underwater acoustic target recognition task, the support vector machine trained on the competitive deep-belief network features can achieve higher classification accuracy with fewer features and achieve 100% true-positive rate with lower false-positive rate than other feature-extraction methods.

## Figures and Tables

**Table 1:** Comparison of proposed methods and traditional feature-extraction methods via classification accuracy and variance.

Methods	Features	Dimension	NMI	Accuracy/%	Variance/×10^−3^
Traditional	MFCC [6]	12	0.315	78.9	5.1
	DMFCC [6]	12	0.184	73.1	5.8
	DDMFCC [6]	12	0.177	71.8	5.6
	Waveform [1,2]	8	0.307	73.9	9.2
	Auditory [5]	24	0.190	75.2	8.3
	Wavelet [3,4]	14	0.269	76.3	7.4
CDBN	Layer1	50	0.392	80.6	3.9
	Layer2	50	0.554	86.7	3.7

## Abbreviations

The following abbreviations are used in this manuscript:

RBM	restricted Boltzmann machine
DBN	deep-belief networks
CRBM	competitive restricted Boltzmann machine
CDBN	competitive deep-belief networks
SVM	support vector machine
MFCC	Mel-frequency cepstral coefficients
DMFCC	first-order differential Mel-frequency cepstrum coefficients
DDMFCC	second-order differential Mel-frequency cepstrum coefficients
DFT	discrete Fourier transforms
NMI	normalized mutual information
ROC	receivers operating characteristic
AUC	area under ROC curve

## References

[1] Meng Q., Yang S. (2015). A wave structure based method for recognition of marine acoustic target signals. J. Acoust. Soc. Am..

[2] Meng Q., Yang S., Piao S. (2014). The classification of underwater acoustic target signals based on wave structure and support vector machine. J. Acoust. Soc. Am..

[3] Azimi-Sadjadi M.R., Yao D., Huang Q., Dobeck G.J. (2000). Underwater target classification using wavelet packets and neural networks. IEEE Trans. Neural Netw..

[4] Wei X., Gang-Hu L.I., Wang Z.Q. (2011). Underwater Target Recognition Based on Wavelet Packet and Principal Component Analysis. Comput. Simul..

[5] Yang L.X., Chen K.A., Zhang B.R., Liang Y. (2014). Underwater acoustic target classification and auditory feature identification based on dissimilarity evaluation. Acta Phys. Sin..

[6] Zhang L., Wu D., Han X., Zhu Z. (2016). Feature Extraction of Underwater Target Signal Using Mel Frequency Cepstrum Coefficients Based on Acoustic Vector Sensor. J. Sens..

[7] Tuma M., Rørbech V., Prior M.K., Igel C. (2016). Integrated Optimization of Long-Range Underwater Signal Detection, Feature Extraction, and Classification for Nuclear Treaty Monitoring. IEEE Trans. Geosci. Remote Sens..

[8] Yang H., Gan A., Chen H., Pan Y. Underwater acoustic target recognition using SVM ensemble via weighted sample and feature selection. Proceedings of the International Bhurban Conference on Applied Sciences and Technology.

[9] Goodfellow I., Bengio Y., Courville A. (2016). Deep Learning.

[10] Yang H., Shen S., Yao X., Han Z. (2017). Underwater Acoustic Target Feature Learning and Recognition using Hybrid Regularization Deep Belief Network. Xibei Gongye Daxue Xuebao/J. Northwest. Polytech. Univ..

[11] Shen S., Yang H., Han Z., Shi J., Xiong J., Zhang X. Learning robust features from underwater ship-radiated noise with mutual information group sparse DBN. Proceedings of the INTER-NOISE and NOISE-CON Congress and Conference.

[12] Hinton G.E. (2012). A Practical Guide to Training Restricted Boltzmann Machines. Momentum.

[13] Lecun Y., Bengio Y., Hinton G. (2015). Deep learning. Nature.

[14] Sarikaya R., Hinton G.E., Deoras A. (2014). Application of Deep Belief Networks for natural language understanding. IEEE/ACM Trans. Audio Speech Lang. Process..

[15] Raina R., Battle A., Lee H. Self-taught Learning Transfer Learning from Unlabeled Data. Proceedings of the 24th International Conference on Machine Learning.

[16] Erhan D., Bengio Y., Courville A., Manzagol P.A., Vincent P., Bengio S. (2010). Why Does Unsupervised Pre-training Help Deep Learning?. J. Mach. Learn. Res..

[17] Haykin S. (2009). Neural Networks and Learning Machines.

[18] Erhan D., Bengio Y., Courville A., Vincent P. (2009). Visualizing Higher-Layer Features of a Deep Network.

[19] Das A., Kumar A., Bahl R. (2013). Marine vessel classification based on passive sonar data: the cepstrum-based approach. IET Radar Sonar Navig..

[20] Hinton G.E. (2008). Visualizing High-Dimensional Data Using t-SNE. Vigil. Christ..

[21] Yang H., Shen S. (2016). The Feature Selection of Pattern Recognition.

